# The CANadian Pediatric Weight management Registry (CANPWR): lessons learned from developing and initiating a national, multi-centre study embedded in pediatric clinical practice

**DOI:** 10.1186/s12887-018-1208-6

**Published:** 2018-07-19

**Authors:** Katherine M. Morrison, Geoff D. C. Ball, Josephine Ho, Pam Mackie, Annick Buchholz, Jean-Pierre Chanoine, Jill Hamilton, Anne-Marie Laberge, Laurent Legault, Lehana Thabane, Mark Tremblay, Ian Zenlea

**Affiliations:** 1 Department of Pediatrics, McMaster University, Hamilton, ON Canada; 2 Population Health Research Institute, McMaster University, Hamilton, ON Canada; 3 Department of Pediatrics, University of Alberta, Edmonton, AB Canada; 4 Department of Pediatrics, University of Calgary, Calgary, AB Canada; 5 Children’s Hospital of Eastern Ontario, Ottawa, ON Canada; 6 Department of Pediatrics, University of British Columbia, Vancouver, BC Canada; 7 The Hospital for Sick Children, Toronto, ON Canada; 8 Department of Pediatrics, CHU Ste Justine, Montreal, QC Canada; 9 Department of Pediatrics, McGill University, Montreal, QC Canada; 10 Credit Valley Hospital, Mississauga, ON Canada

**Keywords:** Childhood obesity, Treatment, Weight management, Pediatric, Research methodology, Cohort study, Cardiometabolic health outcomes

## Abstract

**Background:**

There is increasing recognition of the value of “real-world evidence” in evaluating health care services. Registry-based, observational studies conducted in clinical settings represent a relevant model to achieve this directive. Starting in 2010, we undertook a longitudinal, observational study (the CANadian Pediatric Weight management Registry [CANPWR]), which is embedded in 10 multidisciplinary, pediatric weight management clinics across Canada. The objective of this paper was to share the lessons our team learned from this multi-centre project.

**Methods:**

Data sources included a retrospective review of minutes from 120 teleconferences with research staff and investigators, notes taken during clinical site visits made by project leaders, information from quality control processes to ensure data accuracy and completeness, and a study-specific survey that was sent to all sites to solicit feedback from research team members (*n* = 9). Through an iterative process, the writing group identified key themes that surfaced during review of these information sources and final lessons learned were developed.

**Results:**

Several key lessons emerged from our research, including the (1) value of pilot studies and central research coordination, (2) need for effective and regular communication, (3) importance of consensus on determining outcome measures, (4) challenge of embedding research within clinical practice, and (5) difficulty in recruiting and retaining participants. The sites were, in spite of these challenges, enthusiastic about the benefits of participating in multi-centre collaborative studies.

**Conclusion:**

Despite some challenges, multi-centre observational studies embedded in pediatric weight management clinics are feasible and can contribute important, practical insights into the effectiveness of health services for managing pediatric obesity in real-world settings.

**Electronic supplementary material:**

The online version of this article (10.1186/s12887-018-1208-6) contains supplementary material, which is available to authorized users.

## Background

There is evidence to support the effectiveness of family-centred, multi-disciplinary health services for managing pediatric obesity [1, 2]; however, the impact on weight status is modest. Furthermore, there is limited information available on which models of health care delivery are most effective at improving weight and health and for which populations. Given the identified need to improve our evaluation of outcomes in the Canadian pediatric weight management context, our team of clinical researchers developed the CANadian Pediatric Weight management Registry (CANPWR) [3].

CANPWR is a prospective, national, multi-centre, observational cohort study created to evaluate the individual-, family-, and program-level determinants of *(i)* health outcomes (cardiometabolic health and health related quality of life) at baseline, *(ii)* change in health outcomes over a 3-year period, and *(iii)* attrition from multidisciplinary pediatric weight management clinics located across Canada. Results from our pilot study, which included five clinical sites, were reported previously [4]. The main study [3] is ongoing and was designed to enroll 1600 2–17 year-olds with overweight or obesity from 10 clinics over three years. CANPWR includes the systematic collection of a minimal dataset with the intention of documenting the effectiveness of therapies in real-world clinic settings and enhancing our understanding of which children are more likely to benefit from specific interventions [5]. In recent years, similar registries dedicated to the management of pediatric obesity have been undertaken in the US [6], Sweden [7] and Germany [8, 9]. The experience and insights gained in undertaking these projects, including CANPWR, have the potential to strengthen future registry studies as well as inform the structure and delivery of health services for managing pediatric obesity. The purpose of this paper was to document and share the key lessons that we learned from this multi-centre project.

## Methods

### The history of CANPWR

Our study began as a pilot (2010–2012) that was funded by the Canadian Institutes of Health Research (CIHR) through the Canadian Network and Centre for Trials Internationally program (details available at: www.cannectin.ca). As a pilot, CANPWR included five Canadian tertiary-level, multidisciplinary, pediatric weight management clinics (BC Children’s Hospital in Vancouver, BC; Stollery Children’s Hospital in Edmonton, AB; Children’s Hospital of Eastern Ontario in Ottawa, ON; CHU-Sainte Justine in Montreal, QC; McMaster Children’s Hospital in Hamilton, ON). Data collection was supported by a central coordinating site (Population Health Research Institute, McMaster University, Hamilton, ON). Our pilot study was designed to assess a number of factors that would inform a larger-scale study, including *acceptability* (e.g.*,* Can sites agree on a core set of variables and measurement protocols for data collection? Does data collection for research purposes burden or complement data capture for clinical purposes?) and *feasibility* (e.g.*,* Are sites able to successfully enrol participants into CANPWR from the sample of boys and girls referred to the clinics? Are sites able to retain participants to enable longitudinal research data collection and analysis, regardless of whether individuals discontinue clinical care for weight management?). Data collected during our pilot were compared and contrasted with normative Canadian data, which highlighted the increased health risks present in children referred for weight management [4].

Building on our pilot experience and supported by a CIHR operating grant received in 2012 (Principal Investigator: KMM), CANPWR expanded to eight sites (original five sites, plus The Hospital for Sick Children in Toronto, ON; ICAN clinic in Toronto, ON; Montreal Children’s Hospital in Montreal, QC). Two newly established clinics (Alberta Children’s Hospital in Calgary, AB; Trillium Health Partners in Mississauga, ON) joined CANPWR in 2016, which brought the total number of sites to 10; subsequently, one site (ICAN clinic) became inactive in 2016 due to changes in the clinical practice.

Monthly teleconferences with research coordinators and investigators were recorded and summarized in minutes. For this manuscript, the research coordinator reviewed these minutes, extracted key themes for each call and summarized the main themes. Based on this, a survey was developed and sent to investigators and research coordinators at each site asking if all key processes and challenges of conducting the study were included (Additional file 1). The final edited survey was circulated electronically to team members at the CANPWR clinics between June and August, 2016. Responses were received from team members at all nine active sites. With these data (see Table 1) and supplemented by information from quality control processes referred to in Lesson 4, our manuscript authorship group summarized the findings as lessons. They used an online communication tool (www.slack.com; SLACK Offices, Vancouver, BC) to facilitate communication and host virtual meetings to author our manuscript. A summary of our results, categorized as lessons learned, is provided below.

**Table 1:** Summary of survey responses and corresponding lessons learned from research team members at pilot and main study sites

	Pilot Study (*n* = 4 sites; % YES)	Main Study (*n* = 9 sites; % YES)	Comments on lessons learned
Lesson 1: Site-specific regulatory requirements can be managed effectively through the use of a pilot study and central coordinating centre.			
How much time did it take for Research Ethics Board (REB) approval?	Mean number of days: 133	Mean number of days: 38	REB approval was faster in the Main Study.
			REB approvals from other sites enabled expedited reviews for subsequent sites.
			The Main Study included a final and refined protocol, so fewer amendments were required over time.
Did your REB require participants to complete a *consent to contact* prior to study recruitment?	0%	33%	
Lesson 2: Effective team communication is essential for study coordination and conduct.			
Did you experience challenges with recruitment?	75%	66%	Pilot Study sites shared recruitment strategies with Main Study sites.
			Hiring and training new research staff across study sites introduced gaps in recruitment and follow-up.
Lesson 3: Improving clarity and gaining consensus on measures can be time-consuming, but can also enhance study and data quality.			
Were questions of the family difficult for the sites to acquire?	75%	33%	Questionnaires and measures were refined and consensus reached for important data elements, allowing less difficulty with data collection in main study.
Were the questions on family eating patterns challenging for clinical staff or families?	50%	22%	
Lesson 4: Integrating research with clinical practice can create logistical and operational challenges.			
Was the medical history questionnaire difficult to complete?	75%	33%	In the main study, sites were encouraged to integrate research questions and laboratory tests with clinical practice to make it easier to collect data consistently.
Was the physical exam difficult to complete?	25%	22%	When data were not collected during a clinic visit it was difficult to obtain later.
Were labs results difficult to collect?	25%	11%	Incorporating CRF into clinical care helped with data collection.
			Increased length of CRF to accommodate health outcomes beyond cardiometabolic may have contributed to the observation that there was difficulty in entering data for the Main Study that was not present in the pilot.
Were families able to complete all questionnaires at their first visit?	0%	22%	
Did your first encounter with the family occur at the time of a clinic visit?	100%	77%	
Did you encounter difficulty in entering data?	0%	33%	
Did you experience challenges in collecting clinical data that had been harmonized for the study?	0%	55%	
Were the CANPWR CRF (Case Report Forms) used for clinical purposes, too?	25%	55%	
Most common reasons for study participants choosing to enroll in CANPWR?	-To help others		
	-To improve weight management program		
Clinicians opinions on best reasons to participate in CANPWR?	-Long-term family interactions		
	-Linking weight management programs nationally		
Lesson 5: Study recruitment can be slow; retention is impacted by clinic attrition.			
Did families first learn about CANPWR from clinical team members?	100%	100%	In both the Pilot and Main Studies, clinical staff initially approached families about the study and then connected them with the research coordinators for further details.
Most common reason for families not to agree to recruitment			Lack of time was most commonly noted by the families as a reason not to participate
Did you have difficulty tracking participants over time?	50%	77%	The Main Study extends to three years following the baseline assessment. Sites reported challenges tracking participants (e.g.*,* no longer in clinic; frequent *no-shows* to appointments).

## Results

### Lesson 1: Site-specific regulatory requirements can be managed effectively through the use of a pilot study and central coordinating Centre

The observed challenges of conducting multi-site clinical trials [10] are also relevant to observational studies such as CANPWR. As highlighted, specific requirements and length of time for approval from research ethics boards can vary site-to-site. As recommended, for our pilot study, we first sought and received research ethics approval at our central coordinating site in Hamilton, ON. The time that each site required to prepare and submit the application to their respective Research Ethics Board (REB) varied considerably (maximum difference of by up to 40 weeks). This delay occurred, in part, due to the requirement of one REB that the documents be translated into both English and French. This step was completed after the wording of the consent form and case report form (CRF) in English had been approved by the other REBs. After approval at the central coordinating site, the time from submission to approval at the other pilot sites varied from 5 to 17 weeks. As we transitioned from the pilot to main study, sites that were part of the original CANPWR pilot study had their REB applications approved in 2 to 10 weeks. The five sites that joined CANPWR for the full study had initial review periods of 1.5 to 12 weeks.

In addition to timelines, REB requirements influenced other study aspects including the type of data that could be collected and transmitted electronically to the central data centre. This was a result of varying definitions of personal identifying information and varying procedures for accessing clinical data for research. REB requirements also influenced study recruitment as three sites required clinicians to serve as intermediaries between families and our research team members, which included clinicians administering a *consent to contact* form with families. In other words, families were required to provide their written consent to be contacted by the research team. Because some site leaders perceived that this step might enhance recruitment, the consent to contact step was added at two additional sites. Other site-specific institutional regulations introduced variability in start-up time, including contract negotiations for accessing clinical data, data ownership, and budget. For instance, CANPWR research funds were received from the CIHR and held at our coordinating site in Hamilton. Individual contracts were then prepared with each site to transfer funds based on site-specific study activities (e.g.*,* recruitment, data collection). At one site, contracts were required with both the academic institution and the regional health authority where the clinic was located, a requirement that delayed study initiation.

### Lesson 2: Effective team communication is essential for study coordination and conduct

One of the operational aspects of CANPWR includes monthly teleconference meetings that are led by investigators from our coordinating site in Hamilton. Each month, separate coordinator and investigator teleconferences (duration: 1 h) are held to discuss practical, day-to-day issues as well as broader, academic topics, respectively. This communication has been complemented by an average of one site visit per site so far, made by coordinating site team members to support individual sites and encourage adherence to study policies and procedures. A second site visit is envisioned. These meetings also provided contextual information, serving to highlight the clinic- and research-related variability between sites. Teleconferences and site visits also enabled data management strategies and kept sites accountable to and engaged with all study team members.

The sites varied in their access to a trained research coordinator, especially at study start-up and staff turnover has been common. Three sites enlisted the help of students as either volunteer helpers or research assistants. This created exceptional learning and teaching opportunities as well as reduced costs. Student activities were always overseen by research staff and the implemented quality control measures ensured high quality data. Some challenges introduced by the use of students included frequent turn-over and less flexible schedules. Thus research staff had to organize recurrent training sessions. Ongoing communication with an available central coordinator through both scheduled and ad hoc communications helped to minimize the impact of staff and student turnover.

### Lesson 3: Improving clarity and gaining consensus on outcome measures can be time-consuming, but can also enhance study and data quality

An initial goal of CANPWR was to be the first harmonized, evidence-based registry to identify the key determinants of weight change in pediatric weight management clinics across Canada. The CANPWR investigators designed measures based on the best available evidence at the time. Where strong empirical evidence was lacking, expert, group-level consensus was necessary for measure development. The initial CANPWR measures set was designed along these principles [3]. Through implementation in the pilot sites, we realized that several questions were burdensome and impractical. For example, questions relating to puberty assessment by physician evaluation were discontinued due to challenges in collecting these data. Another example included a narrow focus on cardiometabolic health outcomes in our pilot, so the Main Study included additional data collection on mental health and health-related quality of life. Consensus on unclear definitions of data elements was reached through discussion with research and clinical team members across study sites, facilitated by our monthly teleconferences. A record of all discussions and decisions was maintained by our central research coordinator (PM).

### Lesson 4: Integrating research with clinical practice can create logistical and operational challenges

Although integrating research with clinical practice can be beneficial, there are challenges that can arise. For instance, clinicians have busy schedules. Because they are funded to deliver health services, their interest in research can vary. If there is a lack of interest or attention to details, participant data may be missed. In some clinics, having physical space dedicated to research staff can be limited, which can be sub-optimal for participants and families when collecting data. To mitigate these challenges, CANPWR was embedded into clinical practices, which minimized the time needed by clinicians to identify potential participants, enhanced the feasibility of collecting outcome data, and allowed clinicians to focus on caring for their patients while researchers collected the required data [11].

As CANPWR did not require participating sites to modify their clinical practices, variation in the timing and pace of data collection occurred. The 6-month visit was scheduled 6 months after the date the clinical team considered the beginning of the intervention. This may have been beyond 6 months from study recruitment if there were delays in commencing group sessions. To enable flexibility for study participants, the window for conducting the annual study visits was set broadly (6 months on either side of the calculated date).

To ensure the data used to support the research efforts were valid and secure, CANPWR required a robust data management system and related practices. The CANPWR pilot sites learned that integrating the CRF into routine practice improved data collection. Integration of CANPWR data elements into the workflow of clinic visits was accomplished using paper or electronic data collection forms that were shared among sites and modified by each site to meet their local needs. The study sites that integrated the study case report forms into their clinical practice found that, data elements were more consistently collected and available. The requisite data was then extracted from the clinical record and transferred (online) into the electronic case report form. The specific location of the clinical data extracted by each site for each variable on the study CRF was identified at study start-up and reviewed by the central coordinator during the site visit. Sites were expected to use a paper copy to extract the data from the clinical chart as an intermediate step in order that this could be used for verification if the data was questioned through the routine quality control checks either at the time of data entry if outside the variable limits set or in monthly quality control reports.. Using this system, few challenges were identified in entering data into the study web-based data application.

The coordinating centre implemented monthly performance reports to track incomplete data. Reports were used to identify performance gaps, monitor changes over time, and support continuous quality assurance. These reports were valuable to ensure data completeness.

### Lesson 5: Study recruitment can be slow; retention is impacted by clinic attrition

The ability of the research team to contact participants is influenced by availability and number of research team members, relationship between researchers and clinic team members, number of other ongoing or competing studies at each clinic, clinic flow, and volume of eligible participants. Researchers commented that recruitment was challenging, in part because potential participants were too tired or overwhelmed to want to hear about the research study or complete the informed consent process after a long clinic assessment. Participants’ time pressures and coordination with clinical staff introduced challenges. All sites recruited more slowly than investigators anticipated at study outset. Recruitment was reviewed on monthly teleconference calls to recognize the site personnel for their work, collectively problem-solve challenges, and in some cases, encourage healthy competition (peer pressure) among sites**.** Additional strategies to improve recruitment included the use of a consent to contact form for all eligible participants, introducing research coordinators at initial clinic orientation sessions, frequent communication with clinicians at clinic meetings to provide study reminders and updates, and having the research coordinator available at clinic times to answer participants’ and families’ questions.

CANPWR was designed to follow participants for three years, whether or not they were still engaged in clinical care to determine how health outcomes changed once care was completed and to reduce biases introduced by the high attrition rates often seen from weight management clinics [12]. Participants who discontinued attending the clinic, but remained enrolled in CANPWR, were seen at times that accommodated families and the location depended on family preference and space availability (in clinic, in clinic space but out of clinic time or in research space). They were not seen by the clinical team. Continued study participation did not preclude families from re-engaging in clinical care, and our anecdotal experience revealed that for a small number of families, their research engagement facilitated their re-starting clinical care. All laboratory values were shared with the families and their primary care provider. When designing CANPWR, we recognized that including follow-up data collection for three years would be challenging due to high attrition [13] and because follow-up for most childhood obesity treatment studies is ≤24 months [14]. In our communication with families, we were explicit when explaining that their CANPWR participation was separate from their clinical health services; however, at many sites, when families discontinued attending appointments for weight management, it was challenging to engage them in attending CANPWR study visits. When we surveyed our sites, the majority highlighted difficulties in tracking participants longitudinally, noting that increased research personnel time was required as the study progressed to maintain tracking, largely due to attrition from the clinical programs. To mitigate loss to follow-up, the CANPWR investigators gave families unable to attend an in-person visit, the option to complete follow-up study visits by telephone for self-reported measures only (e.g.*,* current health, medication use, health behaviours). While it was intended that this practical option would be effective for collecting data from participants who discontinued clinical care, to date, less than 10% of follow-up visits were carried out that way. Despite having this mode of data collection available for families, attrition remains a substantial issue in our clinics, which highlights the challenges of this issue in successfully managing pediatric obesity [13].

## Discussion

In this report, we highlight a number of lessons we learned in developing and implementing the CANPWR study at 10 multidisciplinary, pediatric weight management clinics located across Canada. By surveying our site investigators and research personnel, we learned lessons that we believe may be of interest and relevance to other clinical researchers, both within and beyond our field of study. These lessons highlight the importance of planning, the value of pilot studies, the critical role of rigorous data collection procedures, and an active central coordinating site when conducting “real-world” studies. Finding the balance between rigorous data collection and the flexibility required to accommodate variable inter-clinic procedures is an important challenge that must be addressed prior to study initiation.

The lessons presented in this manuscript are similar to those reported by investigators involved in other registry studies [7, 9, 15–19]. These lessons include the need to train investigators to be able to properly conduct research [17]. Strategies recommended by others, and employed in CANPWR include incorporation of monthly or bi-monthly meetings, development of standard operating procedures, training of team members in data collection and data entry techniques, and variable definition classification to avoid data discrepancies [16, 19]. We, and others, have also identified the need to be creative and adopt alternative strategies to improve study recruitment and retention [15]. While others [18] have suggested flexibility in study design and statistical analyses to mitigate potential bias from missing data, we have utilized standardized approaches but have built in flexibility in timelines to assist sites in collecting as complete a dataset as possible. Further, we have similarly learned that it is important to have regular meetings to discuss the status of the registry and related projects, that meeting minutes be circulated, and that a running list of projects, papers, and abstract deadlines be maintained [19].

In spite of the challenges, investigators and research teams reported that the opportunity to be part of a national network of clinics was the “best thing about CANPWR”. Prior to CANPWR, some of the investigators had collaborated on smaller-scale studies related to pediatric obesity in Canada [20]; however, CANPWR represented not only the largest research initiative to date, but the topic and scope of the research had a high degree of clinical relevance to the day-to-day health services delivery of multidisciplinary care for managing pediatric obesity at clinics across the country. Since most pediatric weight management clinics in Canada were relatively new when CANPWR began [21], there was a high level of interest and collegiality to work collaboratively. Given that few programs at the time were evaluating their clinical services [21], participating in CANPWR provided a built-in procedure to contribute data to examine more general research questions while offering the ability to examine site-specific data, which could be used locally at the hospital or health system level for resource allocation and decision-making. From a practical perspective, there was a general desire to learn with and from one another, which is likely due, at least in part, to the challenges many clinicians face in providing health services to children and youth (and their families) with obesity.

## Conclusion

Research studies based in “real-world” settings hold promise to illuminate the efficacy of interventions when implemented in a health care setting. Multiple challenges of conducting such studies have been identified and strategies to address them may improve outcomes. Clinical and research teams highlight the value of participating in such studies to their knowledge and practice.

## Additional file

Additional file 1: Survey utilized to collect data from study sites. (DOC 28 kb)

## Abbreviations

CANPWR: CANadian Pediatric Weight management Registry
CIHR: Canadian Institutes of Health Research
CRF: case report form(s)
REB: Research Ethics Board
