# Supplementary material for: The CANadian Pediatric Weight management Registry (CANPWR): lessons learned from developing and initiating a national, multi-centre study embedded in pediatric clinical practice
Source: BMC Pediatr. 2018 Jul 19;18:237. doi: 10.1186/s12887-018-1208-6 (PMC6053829; doi:10.1186/s12887-018-1208-6)
Supplement: Supplementary file 1 — Survey utilized to collect data from study sites. (DOC 28 kb) [file 12887_2018_1208_MOESM1_ESM.doc]

**RECRUITMENT:**

1. Have you experienced any challenges in recruitment? YES NO

If yes, what challenges have you experienced?

2. How do the families first hear about the study (research or clinical staff)?

3. In speaking with families at your site, what reasons have been given for choosing to participate in CANPWR?

4. In speaking with families at your site, what reasons have been given for choosing not to participate in CANPWR?

5. Does your REB require the use of a ‘consent to contact’ form? YES NO

If NO, are you using a consent to contact anyway? YES NO

6. Are you having trouble tracking participants?

E.g. They never return to clinic and are hard to reach YES NO

If yes, please explain:

7. Does the initial encounter with the family occur at the time of a clinic visit? YES NO

If not, how is it arranged?

8. Are you able to complete all CANPWR questionnaires at the initial encounter? YES NO

If no, which forms are completed later and how long is the delay?

**DATA COLLECTION:**

9. Have you incorporated the questionnaires into routine clinical use? YES NO

10. If YES, Please comment on how easy or difficult this was/is?

11. For each of the components of the CRF please answer the following questions:

**CHART REVIEW – history**

Are the questions difficult to complete YES NO

If yes, are there particular questions which are often problematic? And which ones?

Please estimate the time to completion

**CHART REVIEW – Physical exam**

Are the questions difficult to complete YES NO

If yes, are there particular questions which are often problematic? And which ones?

Please estimate the time to completion.

**CHART REVIEW – Laboratory**

Are the questions difficult to complete YES NO

If yes, are there particular questions which are often problematic? And which ones?

Please estimate the time to completion.

**BASELINE ASKED**

Are the questions difficult to complete YES NO

If yes, are there particular questions which are often problematic? And which ones?

Please estimate the time to completion.

**ASKED QUESTIONS – Eating Habits**

Are the questions difficult to complete YES NO

If yes, are there particular questions which are often problematic? And which ones?

Please estimate the time to completion.

**DATABASE:**

12. Do you keep the paper copy of the CRF after entering data into database? YES NO

13. Have you had any difficulty entering the data? YES NO

If yes, please describe.

14. Please estimate the amount of time it takes to enter a baseline visit into the database?

15. Have you experienced any challenges with collecting data harmonized to

the CANPWR methods? YES NO

16. In your opinion: a) What are the best parts of the CANPWR study? b) What are the most challenging parts of the CANPWR study?

17. Any other comments?
